# A Novel Magnetic β-Cyclodextrin-Modified Graphene Oxide and Chitosan Composite as an Adsorbent for Trace Extraction of Four Bisphenol Pollutants from Environmental Water Samples and Food Samples

**DOI:** 10.3390/molecules29040867

**Published:** 2024-02-15

**Authors:** Yichao Gong, Pengyan Liu

**Affiliations:** 1 School of Eco-Environment, Hebei University, Baoding 071000, China; gyichao@126.com; 2 College of Chemical Engineering and Biotechnology, Xingtai University, Xingtai 054001, China

**Keywords:** composite materials, magnetic solid-phase extraction, endocrine-disrupting chemicals, environmental water samples, food samples

## Abstract

In this study, a novel functionalized magnetic composite (MNCGC) for magnetic solid-phase extraction of bisphenols from environmental and food samples was developed, featuring a multistep synthesis with Fe_3_O_4_, chitosan, graphene oxide, and β-cyclodextrin, crosslinked by glutaraldehyde. Characterization confirmed its advantageous morphology, intact crystal structure of the magnetic core, specific surface area, and magnetization, enabling efficient adsorption and separation via an external magnetic field. The optimized MSPE–HPLC–FLD method demonstrated excellent sensitivity, linearity, and recovery rates exceeding 80% for bisphenol pollutants, validating the method’s effectiveness in enriching and detecting trace levels of bisphenols in complex matrices. This approach offers a new avenue for analyzing multiple bisphenol residues, with successful application to environmental water and food samples, showing high recovery rates.

## 1. Introduction

The concept of endocrine-disrupting chemicals (EDCs) was first proposed by Colborn et al. [1] in 1993. These exogenous substances interfere with the human endocrine system, hinder the metabolism of steroid hormones, and cause diseases related to diabetes mellitus, obesity, cardiovascular disease, cancer, and neurotoxicity [1,2]. Bisphenols (BPs) are among the most common and numerous types of EDCs. A typical BP structure contains two phenylhydroxyl groups. BPs are widely used in electronic products, toys, medical equipment, food packaging materials, disposable tableware, and certain plastics and resins, such as epoxy coatings [3,4]. Bisphenol A (BPA) is a representative BP and has one of the highest outputs among products in the world today [5]. Because of the strict international regulations on the production and use of BPA, bisphenol B (BPB), bisphenol F (BPF), and bisphenol AF (BPAF) have been developed as alternatives to BPA. These analogs are similar in chemical structure to BPA and cause stronger endocrine-disrupting effects than BPA [6]. Studies have shown that BPA analogs may also harm or impact reproduction [7], nerves [8], stress and behavior [9], metabolism, and bone development [10]. In addition, they are more harmful to infants and children than to adults [11].

**Figure 1:**
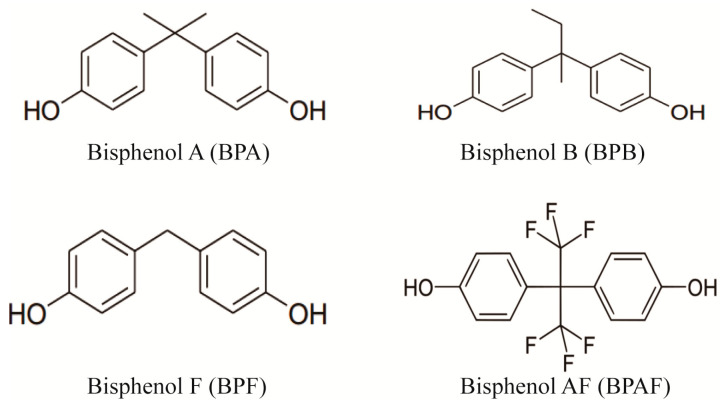
Chemical structural formulas of four major endocrine-disrupting chemicals of bisphenols.

Bisphenol pollutants, detected across various environmental media, exhibit distinct behaviors due to varying Kow values, affecting their distribution in water, sediments, and biological tissues [12,13,14,15,16]. Particularly in China, BPA and BPF have been found in surface and drinking water [12,13,14,15,16,17,18,19], with concerning contamination levels in food, likely due to leaching from packaging [20,21,22]. In addition, the contents of BPF and BPAF detected in some environmental samples were even higher than that of BPA [20]. The chemical structure formulas of four major endocrine-disrupting chemicals of bisphenols is shown in Figure 1. The widespread presence of these pollutants underscores the urgent need for a robust method to analyze their trace residues in environmental and dietary samples.

High-performance liquid chromatography (HPLC) is versatile in analyzing a wide range of compounds, including those with varying polarities and stabilities. It is particularly effective for substances that are less volatile or thermally labile, offering low detection limits and broad applicability. Therefore, HPLC is suitable for determining BP concentrations. Fluorescence detection (FLD) exhibits high sensitivity and favorable selectivity. The performance of FLD is two to three orders of magnitude higher than that of common ultraviolet (UV) detection. FLD only responds to compounds that absorb fluorescence or show fluorescence absorption after derivatization. Most BP pollutants absorb fluorescence. Therefore, FLD not only has high sensitivity to BPs but also can resist interference from impurities in samples [23]. BP pollutants generally remain in trace amounts in environmental water and food samples. Additionally, the matrices of environmental water and food samples are often complicated, precluding direct measurement. Therefore, such samples need to be pretreated before analysis [24]. In recent years, various adsorption-based pretreatment techniques, including solid-phase extraction (SPE), magnetic solid-phase extraction (MSPE), matrix solid-phase dispersion, and solid-phase microextraction, have been developed. MSPE is based on SPE and matrix solid-phase dispersion with the addition of magnetism. This method does not require complicated centrifugal separation during the extraction, enrichment, or elution processes. Rapid separation of the adsorbent from the solution can be achieved simply by applying a magnetic field. In addition, the elution and cleaning procedures for magnetic adsorbents are convenient, enabling easy separation, regeneration, and recycling [25,26,27].

However, the adsorbents used in MSPE pretreatment present problems, such as poor extraction of trace targets, difficult elution, severe matrix interference, poor target selectivity, and poor recycling performance. Selecting a variety of functional monomers for the synthesis of adsorbents is one of the most effective solutions to these problems. Chitosan (CS) is the only natural alkaline polysaccharide and is insoluble in water but soluble in acetic acid and most organic acids. The surface of CS has many amino groups and hydroxyl groups. CS features many unique characteristics, including biocompatibility, biodegradability, nontoxicity, renewable production, and modifiability [28]. Graphene oxide (GO), a graphene derivative, is adorned with oxygenated groups such as carbonyl, hydroxyl, and carboxyl, enhancing its dispersibility and affinity for other molecules via π–π, hydrogen, and hydrophobic bonds [29]. β-Cyclodextrin (β-CD), a cyclodextrin variant, possesses a hydrophobic core and hydrophilic exterior, enabling the formation of stable inclusion complexes with diverse molecules through interactions like hydrophobic, electrostatic, van der Waals forces, dipole–dipole, and hydrogen bonding [30]. β-CD is the most common host because it is readily available and can encapsulate a variety of compounds. However, CD can easily form intermolecular hydrogen bonds, which influence the adsorption effect. Therefore, β-CD-modified silica, graphene, and CS are often used as adsorption materials. Fe_3_O_4_ nanoparticles are usually used as the cores of magnetic adsorption composites [31]. Pure Fe_3_O_4_ nanoparticles are easily oxidized in air, causing problems such as changes in magnetism, reduced dispersion, and even loss of surface active sites [32]. At present, one of the most common modification methods is to coat the surface of Fe_3_O_4_ nanoparticles with SiO_2_ to improve oxidation resistance and dispersion and facilitate surface modification [33]. Notably, no studies have used CS-, GO-, or β-CD-functionalized magnetic nanoadsorbents for the extraction of trace BPA, BPB, BPF, or BPAF from environmental water or food samples.

In this study, a CS-, GO-, and β-CD-functionalized magnetic composite (MNCGC) was developed on the basis of a preparation method involving multiple steps, including a sol-gel reaction, crosslinking, and amidation. Fe_3_O_4_ was synthesized as the magnetic core; CS, GO, and β-CD were used as functional monomers. The synthesized MNCGC was used as an adsorbent in MSPE of BPA, BPB, BPF, and BPAF. The extraction and elution conditions were optimized. Interference tests, a recycling test, methodological evaluation, and real-sample application were conducted under optimal conditions. The MSPE technique was combined with HPLC–FLD, and a method for analyzing trace residues of four BP pollutants in environmental water and food samples was successfully established.

## 2. Results and Discussion

### 2.1. Characterization

**Figure 2:**
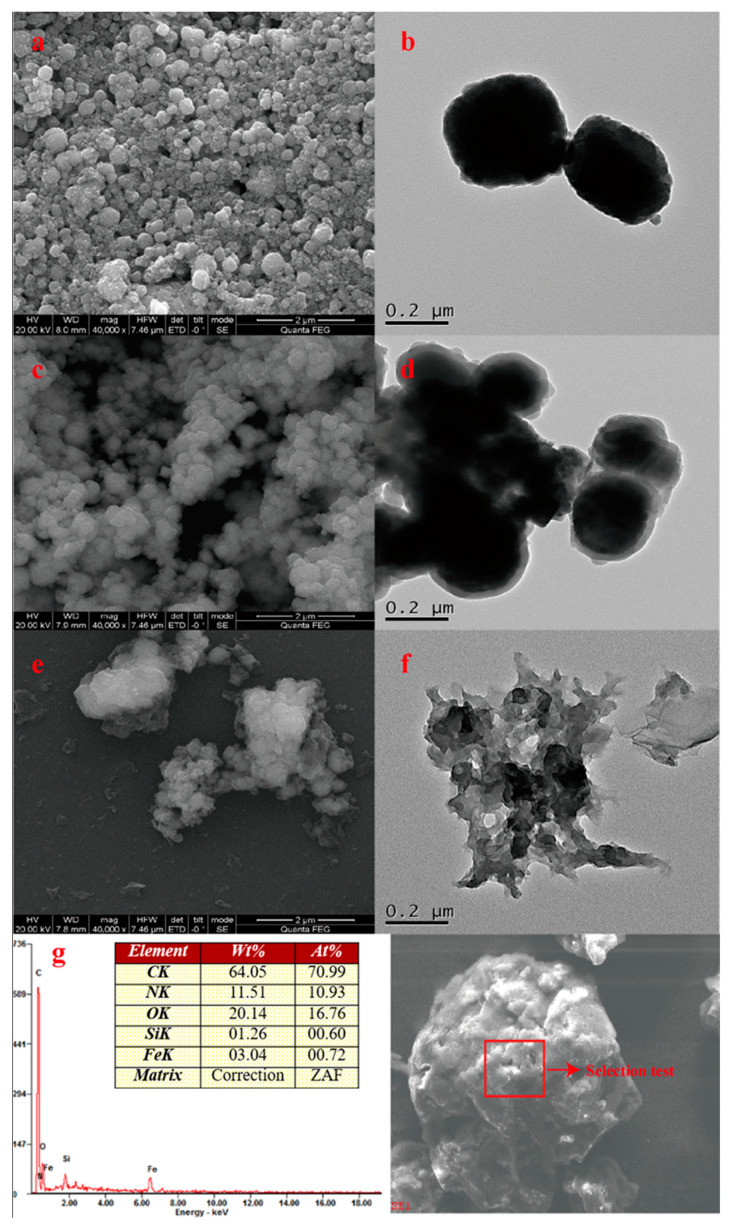
SEM images of Fe_3_O_4_ (**a**); silica−coated Fe_3_O_4_ (**c**); and MNCGC (**e**); TEM images of Fe_3_O_4_ (**b**); silica−coated Fe_3_O_4_ (**d**); and MNCGC (**f**); energy dispersive spectroscopy results for MNCGC (**g**).

Figure 2 shows the SEM and TEM images of Fe_3_O_4_, silica-coated Fe_3_O_4_, and MNCGC, as well as the energy dispersive spectroscopy (EDS) result for MNCGC (Figure 2g). The SEM images are shown on the left (Figure 2a,c,e), and the TEM images are shown on the right (Figure 2b,d,f). As shown in Figure 2a,b, the synthesized Fe_3_O_4_ was approximately spherical and partially agglomerated, with a diameter of approximately 200–300 nm. Figure 2c,d show that the silica-coated Fe_3_O_4_ particles were approximately 5–10 nm larger than the Fe_3_O_4_ particles. After SiO_2_ coating, the surfaces of the particles became smoother, and agglomeration was greatly reduced. It is possible that the SiO_2_ coating not only reduced dipole–dipole interactions between nanoparticles to prevent oxidation and reduce agglomeration but also improved dispersion and stability, which facilitated modification. Figure 2e,f show that the MNCGC surface was wrinkled with large flakes, and the edges were characterized by wrinkled flake structures. These results indicate that CS and GO were successfully introduced. Furthermore, the MNCGC surface was rough. In addition, Figure 2f shows obvious β-CD accumulation on the MNCGC surface. The EDS results for MNCGC (Figure 2g) show that it contained C, N, O, Si, and Fe. The content of C was the highest (64.05%), the content of Si was the lowest (1.26%), and the contents of N, O, and Fe were 11.51%, 20.14%, and 3.04%, respectively. Since CS and GO mainly contain C and N, the contents of C and N in MNCGC were the greatest. Therefore, these results further indicate the successful preparation of MNCGC.

**Figure 3:**
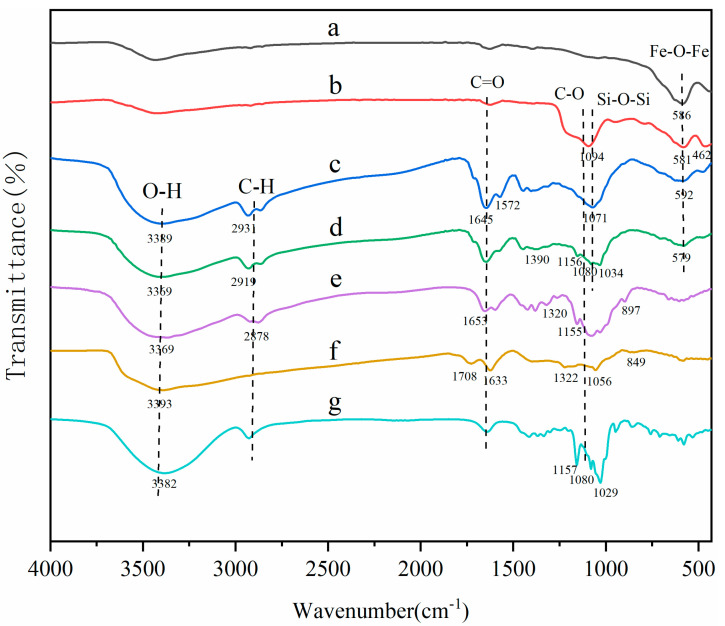
FTIR spectra of Fe_3_O_4_ (**a**), silica−coated Fe_3_O_4_ (**b**), MNCGC before β−CD modification (**c**), MNCGC (**d**), CS (**e**), GO (**f**), and β−CD (**g**).

The surface groups of MNCGC were studied by FTIR (Figure 3). Spectra a, b, c, and d are the FTIR spectra of Fe_3_O_4_, silica-coated Fe_3_O_4_, MNCGC before β-CD modification, and MNCGC, respectively; spectra e, f, and g are reference spectra of CS, GO, and β-CD, respectively. In curve a, the peak at 586 cm^−1^ corresponds to the Fe-O-Fe stretching vibration; in curve b, the peaks at 1094 cm^−1^ and 462 cm^−1^ are the Si-O-Si asymmetric stretching vibration and Si-O stretching vibration, respectively, indicating that SiO_2_ has been successfully coated on the Fe_3_O_4_ surface. In curve e, the peaks between 897 cm^−1^ and 1155 cm^−1^ are the C-O-C stretching vibrations and C-O stretching vibrations, the peak at 1320 cm^−1^ is the C-N stretching vibration, the peak at 3369 cm^−1^ is the O-H stretching vibration, and the heightened peaks at 1653 cm^−1^ and 2878 cm^−1^ suggest the presence of N-H bonds. The peak at 1633 cm^−1^ in curve f is the C=C stretching vibration, the peak at 1708 cm^−1^ is the C=O stretching vibration, and the peaks between 849 cm^−1^ and 1322 cm^−1^ are the epoxide stretching vibrations. In curve c, the same functional groups can basically be found around the corresponding wavenumbers, but the amide reaction between the amino groups of CS and the carboxyl groups of GO leads to the disappearance of the C=O stretching vibration peak at 1708 cm^−1^, and the appearance of N-H bending vibration characteristic peaks at 1572 cm^−1^ and C=O stretching vibration characteristic peaks at 1645 cm^−1^. Compared with curve c, the increase in the area of the O-H bending vibration peak at 1390 cm^−1^ in curve d is attributed to the presence of C-H/O-H bending vibration peaks, coupled C-O/C-C bending vibration peaks and O-H stretching vibration peaks, the typical characteristic peaks of curve g at 1034 cm^−1^, 1080 cm^−1^, and 1156 cm^−1^, and the O-H stretching vibration peak at 3369 cm^−1^ has shifted to the red compared with the corresponding vibration peak of curve c (3389 cm^−1^) and the free O-H vibration peak (3700 cm^−1^), all of which indicate that MCGC has been successfully prepared, and there exist strong hydrogen bonding, hydrophobic interactions, and van der Waals forces in the integration of β-CD and Fe_3_O_4_ into the composite.

**Figure 4:**
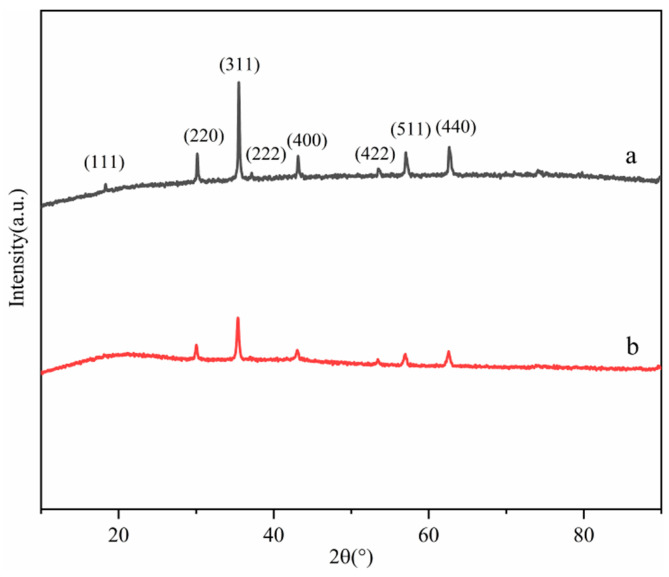
XRD patterns of Fe_3_O_4_ (**a**) and MNCGC (**b**).

The XRD patterns of Fe_3_O_4_ (a) and MNCGC (b) are shown in Figure 4. Both XRD patterns exhibited six characteristic peaks corresponding to Fe_3_O_4_ at 30.128° (220), 35.544° (311), 43.157° (400), 53.55° (422), 57.063° (511), and 62.699° (440), which matched the standard card of Fe_3_O_4_ (JCPDS card no. 89-0950). These results indicate that Fe_3_O_4_ was not lost during the preparation of MNCGC. However, the peak intensity of Fe_3_O_4_ decreased after the introduction of CS, GO, and β-CD, indicating that the content of Fe_3_O_4_ decreased in MNCGC.

**Figure 5:**
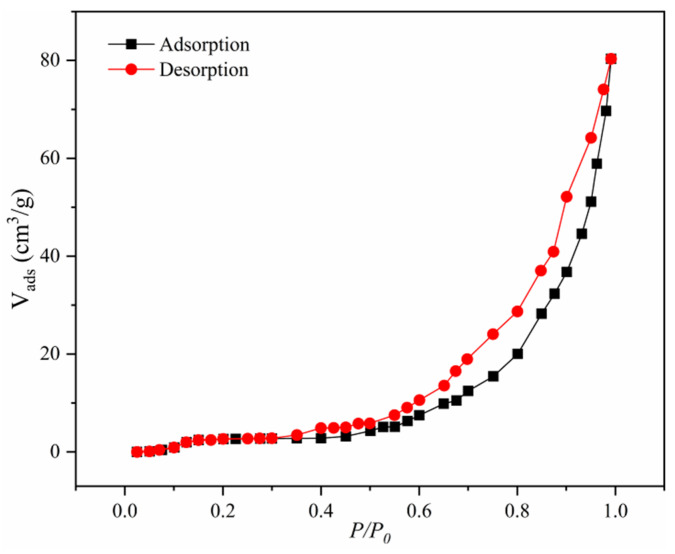
Nitrogen adsorption–desorption isotherm of MNCGC.

The BET nitrogen adsorption–desorption method was used to determine the specific surface area and pore size of MNCGC. The results are shown in Figure 5. MNCGC exhibited a type I/IV isotherm, indicating that the composite had microporous and mesoporous structures. BET analysis showed that the specific surface area of MNCGC was approximately 91.83 m^2^/g and that the average pore size was 12.08 nm.

**Figure 6:**
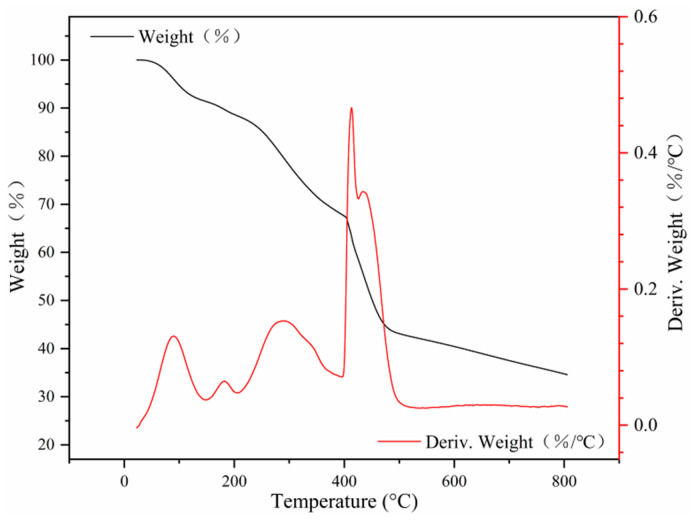
TGA curve of MNCGC.

The content of organic compounds on the surface of the Fe_3_O_4_ core in MNCGC was analyzed by TGA. As shown in Figure 6, the weight loss of MNCGC tended to remain stable above 500 °C, suggesting that only heat-resistant Fe_3_O_4_ remained. The content of organic compounds in the Fe_3_O_4_ core was approximately 49.91%. Below 100 °C, the weight of MNCGC decreased relatively slowly, but the derivative of the weight percentage gradually increased. This result was attributed to the loss of residual water on the MNCGC surface. Oxygen-containing groups decomposed between 107 °C and 237 °C. The decomposition of CS, GO, and β-CD mainly occurred between 251 °C and 471 °C. CS, GO, and β-CD accounted for approximately 45.33% of the total weight, while SiO_2_ accounted for approximately 4.58%.

**Figure 7:**
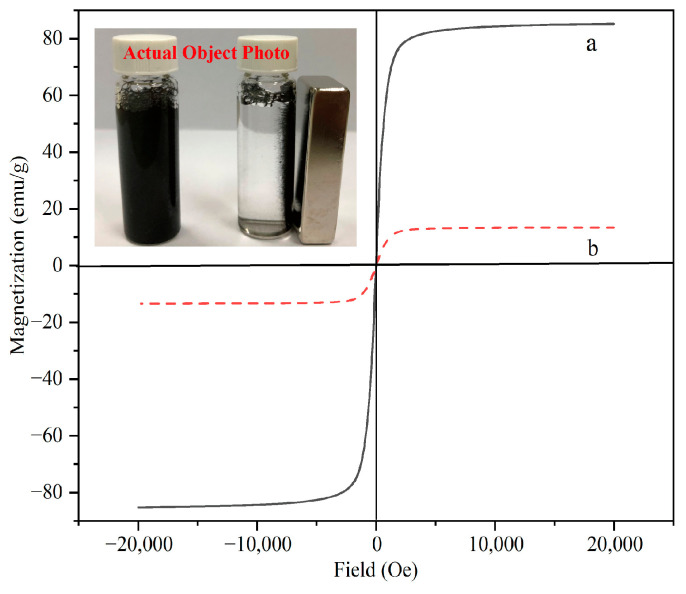
Magnetic hysteresis of Fe_3_O_4_ (**a**) and MNCGC (**b**).

The magnetic properties of the materials were measured by vibrating sample magnetometry (VSM) at room temperature. Figure 7 shows the hysteresis curves of Fe_3_O_4_ (a) and MNCGC (b). The maximum saturation magnetization values were 85.3 emu/g (Fe_3_O_4_) and 16.2 emu/g (MNCGC). Although the level of magnetization decreased after the introduction of CS, GO, and β-CD, the remaining magnetization was still sufficient for MNCGC to be separated by an external magnetic field (inset in Figure 7).

### 2.2. Optimization of MSPE

#### 2.2.1. Optimization of Adsorbent Amount

**Figure 8:**
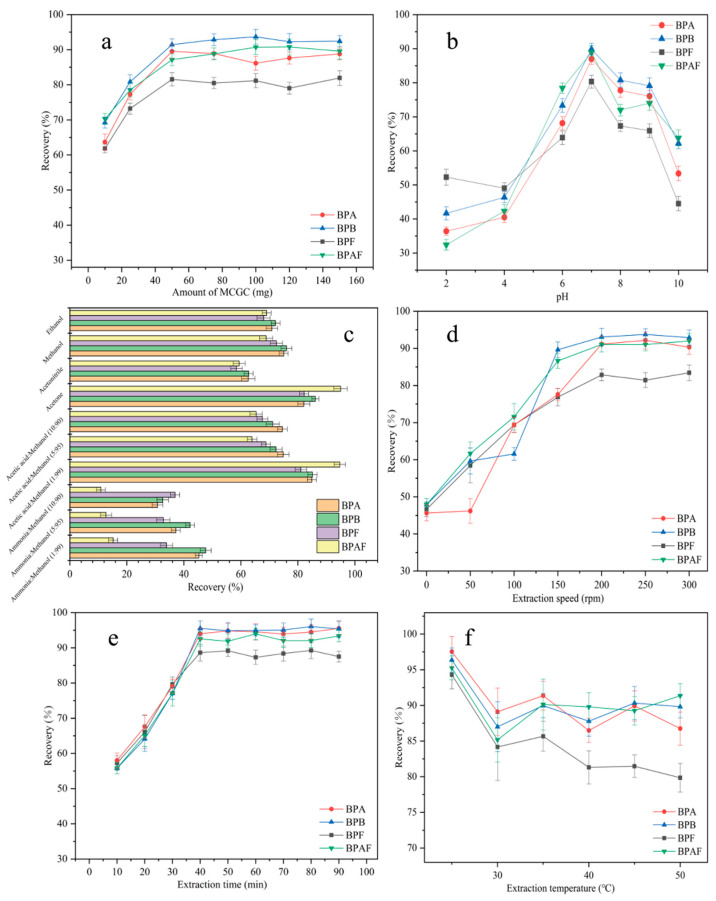
Optimization of adsorbent mass (**a**); optimization of sample pH (**b**); optimization of eluent (**c**); optimization of extraction speed (**d**); optimization of extraction time (**e**); optimization of extraction temperature (**f**).

Generally, the amount of adsorbent plays a key role in the MSPE process. To achieve the highest extraction efficiency and maximize the performance of MNCGC, the effect of MNCGC mass (from 10–150 mg) on the recovery of the four BP pollutants was investigated (Figure 8a). As the mass of MNCGC increased, the recovery of all four BP pollutants increased. The recovery of the four BP pollutants reached maximum values at 50 mg MNCGC. At this time, the percent recovery of BPA, BPB, BPF, and BPAF was 81.6%, 89.5%, 91.4%, and 87.2%, respectively. As the MNCGC mass continued to increase, the recovery did not substantially change and remained stable. Therefore, 50 mg was selected as the mass of the extraction adsorbent.

#### 2.2.2. Optimization of Sample pH

pH generally plays a crucial role in MSPE. pH can affect the form of the target compound in water and the interaction between the target and the adsorbent. The effect of pH (pH = 2–10) on extraction recovery was studied by using 0.1 M NaOH and HCl as pH regulators (Figure 8b). At pH = 7, the recovery of the four BP pollutants was the highest. As the pH increased from 2 to 7, the recovery of the four BP pollutants increased. As the pH increased from 7 to 10, the recovery decreased. BPA, BPB, BPF, and BPAF contain hydroxyl groups, and they can be deprotonated. Their average pKa values are 10.1, 10.0, 10.8, and 9.2. At pH > 7, the BPs are deprotonated and cannot form an inclusion complex with β-CD in MNCGC. Therefore, the recovery of the four BP pollutants was lower at pH > 7 than at pH = 7. However, at pH < 7, although the four BP pollutants are in the molecular state, they can be partially protonated because of the acidic conditions. MNCGC has amino groups on its surface, which cause repulsion between the target and the adsorbent. As a result, the recovery of the four BP pollutants was lower at pH < 7 than at pH = 7. Therefore, the pH of the solution was adjusted to 7 for subsequent experiments.

#### 2.2.3. Optimization of the Eluent

The type of eluent is a key factor affecting the extraction efficiency of MSPE. The four BP pollutants are slightly nonpolar compounds. Additionally, the desorption efficiency can be improved by adding a small amount of ammonia solution or acetic acid to the eluent. Therefore, desorption was performed using common eluents and desorbents, including ethanol, methanol, acetonitrile, acetone, 1% ammonia–methanol solution, 5% ammonia–methanol solution, 10% ammonia–methanol solution, 1% acetic acid–methanol solution, 5% acetic acid–methanol solution, and 10% acetic acid–methanol solution (Figure 8c). Among the four pure eluents, acetone and methanol showed the highest recoveries after elution. However, acetone is a precursor compound and is harmful to the human body. Thus, ammonia solution or acetic acid was added to methanol to prepare mixed desorbents. The elution effects of the mixed desorbents were compared with that of acetone. Acetone and 1% acetic acid–methanol solution had the most favorable desorption effects on MNCGC. Therefore, 1% acetic acid–methanol solution was selected as the eluent for subsequent experiments.

#### 2.2.4. Optimization of Extraction Speed, Extraction Time, and Extraction Temperature

The extraction speed is another important factor affecting MSPE. The effect of extraction speed (0–300 rpm) on recovery was studied (Figure 8d). The extraction speed had a strong impact on recovery. As the extraction speed increased, the recovery increased sharply. The recovery of the four BP pollutants was highest at 200 rpm. As the speed increased further from 200 rpm to 300 rpm, the recovery tended to remain stable. Therefore, 200 rpm was selected as the optimal extraction speed. To optimize the extraction time, the effect of extraction time (10–90 min) on recovery was investigated (Figure 8e). Initially, the recovery of the four BP pollutants gradually increased with time. After 40 min, recovery reached a maximum. As the extraction time continued to increase, the recovery did not change substantially. Therefore, in subsequent experiments, 40 min was selected as the extraction time. The extraction temperature can affect the interaction between the target and the adsorbent, thereby influencing the extraction effect. The effect of extraction temperature (20–50 °C) on recovery was investigated (Figure 8f). Recovery was the highest at 25 °C and decreased as the temperature further increased. Lowering the temperature significantly increased the extraction recovery. Therefore, 25 °C was selected as the optimal extraction temperature.

#### 2.2.5. Optimization of Ion Type and Ionic Strength

**Figure 9:**
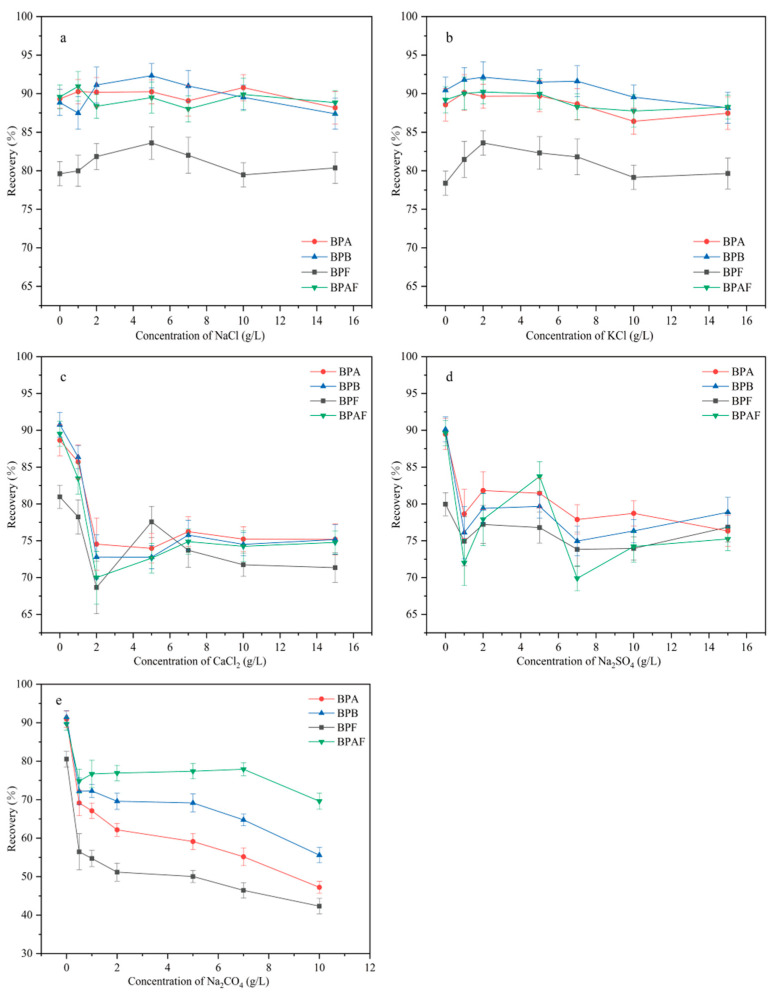
Effect of ion type and ionic bond strength on extraction: (**a**) NaCl; (**b**) KCl; (**c**) CaCl_2_; (**d**) Na_2_SO_4_; (**e**) Na_2_CO_4_.

The ion type and ionic bond strength are important factors that affect MSPE recovery. These factors can affect the solubility of the target compound, the diffusion of the target between the aqueous and solid phases, and the number of active sites on the adsorbent. Therefore, the effect of five inorganic salts (in the concentration range of 0–15 g/L) on extraction was investigated (Figure 9). Because of the salting-out effect, the recoveries of the four BP pollutants first increased and then decreased as the added amount of NaCl and KCl increased. When the concentrations of NaCl and KCl were 5 g/L and 2 g/L, respectively, recovery reached a maximum. As the concentrations of NaCl and KCl continued to increase, the recovery decreased, possibly because the high concentration of ions hindered the diffusion of the target to the adsorbent. As the added amount of CaCl_2_, Na_2_SO_4_, and Na_2_CO_4_ increased, the recovery of all four BP pollutants decreased. This decrease possibly occurred because of the amino and carboxyl groups on the MNCGC surface, which may have caused Ca^2+^ and SO_4_^2−^ to occupy the active adsorption sites of the target, resulting in reduced recovery. CO_4_^2−^ is a weak base that can deprotonate the four BP pollutants. As a result, the BPs not only failed to form an inclusion complex with β-CD but also repelled the adsorbent. Therefore, the addition of Na_2_CO_4_ led to the greatest decrease in recovery. The maximum recovery percentages after NaCl addition and KCl addition were almost the same. Therefore, 2 g/L KCl was added in subsequent experiments.

#### 2.2.6. Optimization of Elution Volume, Elution Speed, Elution Time, and Elution Temperature

**Figure 10:**
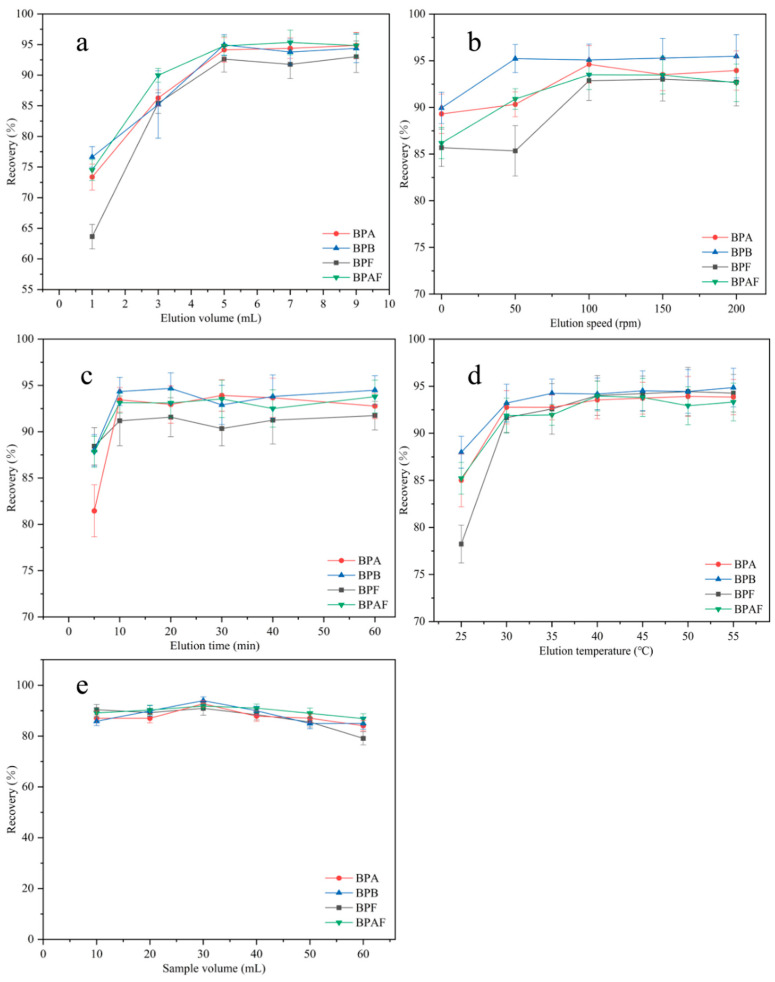
Optimization of elution volume (**a**); optimization of elution speed (**b**); optimization of elution time (**c**); optimization of elution temperature (**d**); selection of sample volume (**e**).

The elution volume was optimized from 1 to 9 mL (Figure 10a). The recovery of the four BP pollutants increased as the elution volume increased. The maximum recovery was reached when the elution volume was 5 mL. As the elution volume continued to increase, the recovery tended to remain stable. Therefore, 5 mL was chosen as the optimal elution volume. The effect of elution speed on recovery was investigated from 0 to 200 rpm (Figure 10b). The recovery of the four BP pollutants increased as the elution speed increased. The maximum recovery of BPB was achieved at an elution speed of 50 rpm, and the maximum recovery of the remaining BPs was achieved at 100 rpm. As the elution speed continued to increase, the recoveries of the four BP pollutants remained basically unchanged. Therefore, 100 rpm was chosen as the optimal elution speed for subsequent experiments. The elution time was optimized in the range of 5–60 min (Figure 10c). The recovery of the four BP pollutants increased with elution time. At 10 min, the recovery was the highest. As the elution time further increased, the recovery remained basically unchanged. To shorten the elution time and improve efficiency, 10 min was selected as the optimal elution time for subsequent experiments. The effect of elution temperature on recovery was investigated from 25 to 55 °C (Figure 10d). The elution of the four BP pollutants was promoted by increasing the temperature. The recovery of BPAF reached a maximum at 40 °C, and the recoveries of the other three BPs peaked at 45 °C. As the elution temperature further increased, the recovery remained basically stable. To ensure the maximum recovery of all four BP EDCs, 45 °C was chosen as the elution temperature for subsequent experiments.

#### 2.2.7. Selection of Sample Volume

The sample volume also affects the extraction efficiency. First, 10, 20, 30, 40, 50, and 60 mL samples of 10 μg/L BP solution were accurately measured. Under optimal conditions, extraction was performed following the MSPE procedure described in Section 3.3, and the results are shown in Figure 10e. In the sample volume range of 10–60 mL, recovery exceeded 80% for all BP pollutants. The maximum recovery was achieved when the sample volume was 30 mL. As the solution volume further increased, recovery decreased. Therefore, 30 mL was selected as the sample volume for quantitative analysis.

### 2.3. Interference Test

Real environmental and food samples have complex matrices that contain multiple components. For example, environmental water contains natural organics, such as humic acid (HA), and most foods contain sugars, such as glucose, fructose, and sucrose. These substances may affect the extraction of BPs by interference and competition. Therefore, the influence of HA and sugars on extraction efficiency must be investigated.

#### 2.3.1. Effect of Humic Acid on Extraction Efficiency

**Figure 11:**
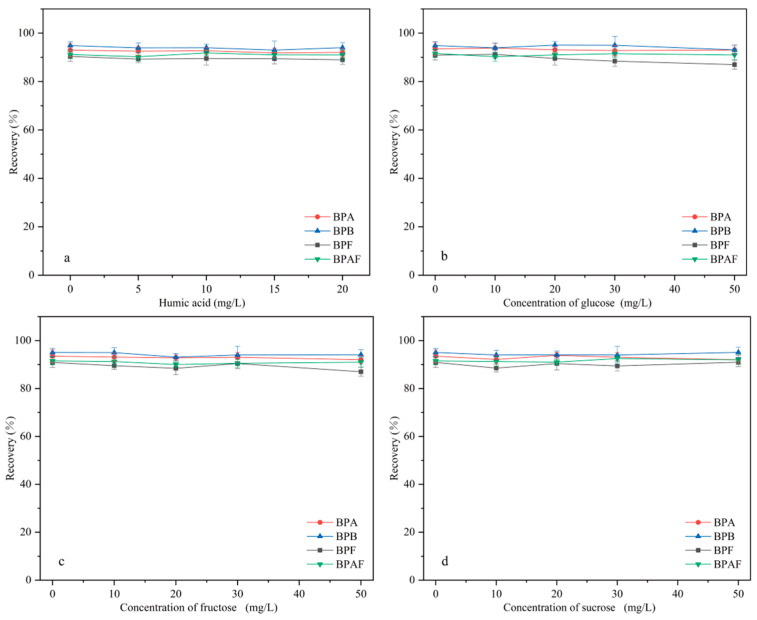
Influence of interference factors on recovery: (**a**) humic acid; (**b**) glucose; (**c**) fructose; (**d**) sucrose.

HA is widely distributed in water bodies. The effect of HA (in the concentration range of 0–20 mg/L) on MNCGC extraction was studied. First, certain amounts of HA were accurately weighed and brought to a 100 mL volume using 10 μg/L BP solution to prepare HA solutions with concentrations ranging from 0 to 20 mg/L. Extraction was performed under the optimal conditions following the MSPE procedure described in Section 3.3, and the results are shown in Figure 11a. Increases in the concentration of HA resulted in almost no effect on the extraction of the four BP pollutants. In addition, studies have reported that the total organic carbon content in environmental waters is generally below 3.5 mg/L [33]. Therefore, the influence of natural organic compounds on the extraction of BP pollutants by MNCGC can be ignored.

#### 2.3.2. Effect of Sugars on Extraction Efficiency

Most foods contain sugars. To investigate the influence of sugars on extraction efficiency, sugars that are often added to foods (glucose, fructose, and sucrose) were used in extraction experiments. First, certain amounts of glucose, fructose, and sucrose were accurately weighed and brought to a 100 mL volume using 10 μg/L BP solution to prepare sugar solutions with concentrations ranging from 0 to 50 mg/L. Extraction was performed under the optimal conditions following the MSPE procedure described in Section 3.3, and the results are shown in Figure 11b–d. The addition of sugar had no significant effect on extraction. Therefore, the influence of sugars on the application of MNCGC to food samples can also be ignored.

### 2.4. Method Validation

The linearity range, limit of detection (LOD), limit of quantification (LOQ), and precision of the MSPE method were further evaluated by applying HPLC–FLD under the optimal conditions. Standard working solutions with concentrations ranging from 0.03 to 100 μg/L were prepared by diluting the standard BP stock solution with ultrapure water. Analysis was performed under the optimal extraction conditions. For each concentration, three parallel runs were performed, and each sample was tested three times. The intraday precision was determined by measuring the 10 μg/L standard solution six times a day (relative standard deviation, RSD, *n* = 6), and the interday precision was determined by measuring the 10 μg/L standard solution for three consecutive days (RSD, *n* = 3). As shown in Table 1, BPA, BPB, BPF, and BPAF showed good linear relationships, and the correlation coefficient (*R*) values were all greater than 0.998. The LOD and LOQ were calculated based on signal-to-noise (S/N) ratios of 3 and 10. The values were 0.01 μg/L and 0.03 μg/L for BPA, 0.01 μg/L and 0.05 μg/L for BPB, 0.02 μg/L and 0.05 μg/L for BPF, and 0.01 μg/L and 0.04 μg/L for BPAF, respectively. The intraday and interday precision values were less than 5.0%.

### 2.5. Recycling Experiment

**Figure 12:**
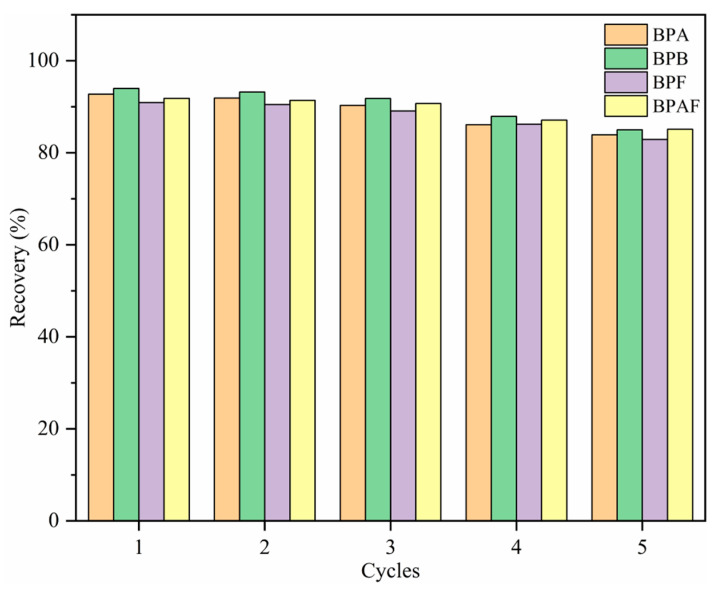
Regeneration capacity of MNCGC after multiple cycles.

To study the regeneration capacity of MNCGC, 30 mL of 10 μg/L BP solution was accurately measured. MSPE was performed under the optimal extraction conditions according to the procedure described in Section 2.3. The extraction–elution process described above was repeated five times, and the results are shown in Figure 12. After five extraction–elution cycles, recovery still exceeded 80% for the four BP pollutants, possibly because MNCGC was prepared from a variety of functional monomers. These results also suggest that MNCGC can achieve high regeneration performance.

### 2.6. Analysis of Real Samples

**Figure 13:**
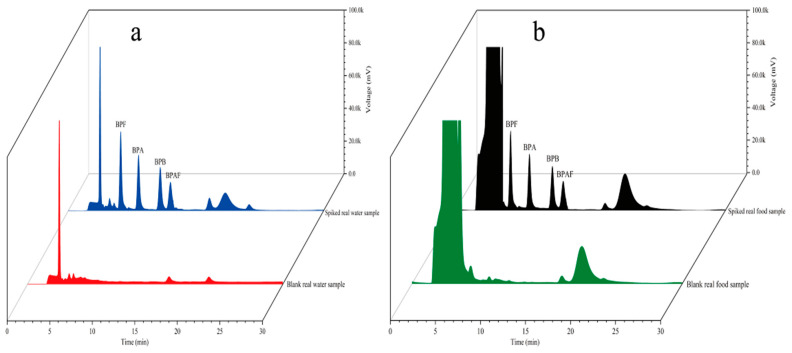
Chromatograms of environmental samples (**a**) and food samples (**b**).

To examine the accuracy of the established method, four environmental water samples and three food samples were analyzed. The coordinates of the environmental water sampling locations are shown in Table 2. Samples S1 and S2 were collected from Fuhe, Baoding, Hebei Province, China; samples S3 and S4 were collected from Baiyangdian, Xiong’an New District, China; and walnut milk, peach juice, and orange juice were purchased from a supermarket in Baoding, Hebei Province, China. The environmental water samples were suction-filtered through 0.45-μm hydrophilic microporous membrane filters to remove sand and soil and then stored in brown glass bottles for later use. The food samples were placed in 50 mL centrifuge tubes and centrifuged at 4000 rpm for 5 min to remove large sediment particles. Then, the samples were filtered through 0.45-μm hydrophilic microporous membrane filters and stored in brown glass bottles for later use. All samples spiked with different concentrations (5 μg/L and 10 μg/L) were analyzed using the established method under the optimal conditions. Each sample was analyzed in parallel three times. The results are shown in Table 3. The chromatograms of the spiked samples and blank samples are shown in Figure 13. Figure 13 shows that the blank samples had basically no interference. Table 3 shows that the recovery of the spiked real samples ranged from 89.1 to 103.7%. The method established in this study clearly has sufficient practicability for the determination of trace residues of the four target BP pollutants in environmental water and food samples.

### 2.7. Comparison of Methods and Adsorption Mechanism

**Figure 14:**
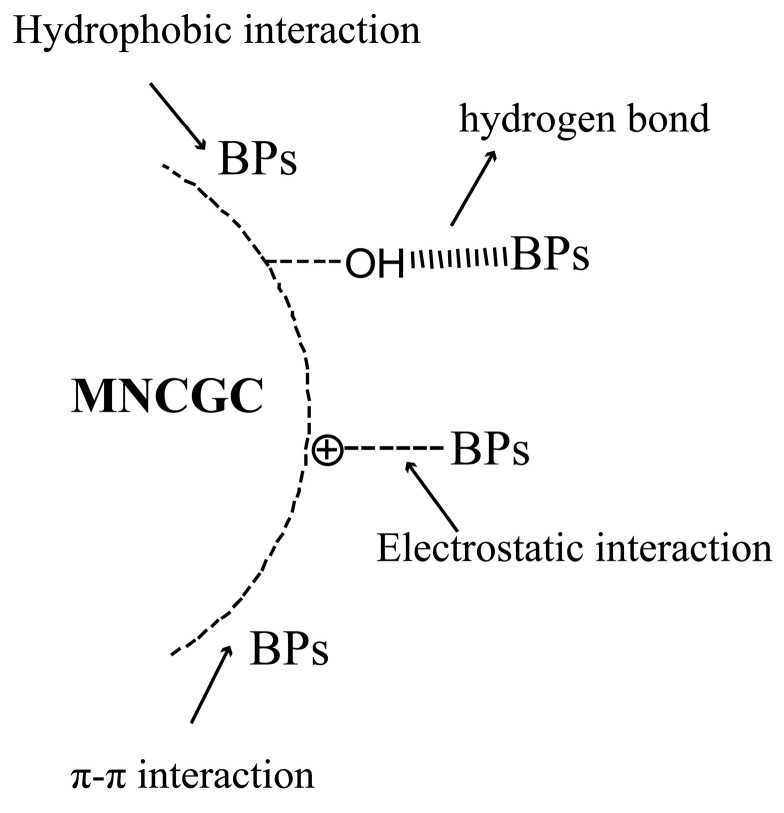
The adsorption mechanism of MNCGC.

The MSPE–HPLC–FLD method developed in this study was compared with other methods reported in the literature for the simultaneous detection of multiple BP pollutants (Table 4). This method had a lower LOD than most of the other methods listed in Table 4. Although the LOD of this method was not as low as that of the MSPE–HPLC–MS method, it was relatively close. Its adsorption mechanism is shown in Figure 14, after CS grafted on MNCGC, the amino and hydroxyl groups on the surface may have electrostatic interaction and hydrogen bonding with the hydroxyl groups in the bisphenol compound, while the grafted GO has a benzene ring-like structure, and is on the edge and base surface. It contains a large number of oxygen-containing groups, which may adsorb bisphenols through π-π attractive force, hydrophobic force and hydrogen bond force. β-CD on the surface of MNCGC has a cavity structure, internal hydrophobicity, and external hydrophilic properties, and it can form a more stable inclusion compound with bisphenol compounds through hydrophobic force. Therefore, the MSPE–HPLC–FLD method features a low LOD and favorable recovery; moreover, this method does not require complicated or time-consuming centrifugal separation. This study provides a new method for analyzing trace residues of multiple BP pollutants in environmental water and food samples.

## 3. Materials and Methods

### 3.1. Reagents and Apparatus

BPA (>99%), BPB (>99%), BPF (>99%), and BPAF (>99%) were purchased from Aladdin Chemistry Co., Ltd. (Shanghai, China). The mixed standard stock solutions (100 μg/L) of BPA, BPB, BPF, and BPAF were stored in the dark at 5 °C. Ferric chloride hexahydrate (FeCl_3_·6H_2_O, 99%), ferrous sulfate heptahydrate (FeSO_4_·7H_2_O, 99%), ammonium solution (NH_3_·H_2_O, 25%), sodium chloride (NaCl, 99%), sodium hydroxide (NaOH, 99%), hydrochloric acid (HCl, 37%), sodium carbonate (Na_2_CO_3_, 99%), calcium chloride (CaCl_2_, 99%), sodium sulfate (Na_2_SO_4_, 99%), and potassium chloride (KCl, 99%) were purchased from Beichen Fangzhen Reagent Factory (Tianjin, China). CS (90% degree of deacetylation), β-CD (99%), and glutaraldehyde (50% in water) were purchased from Titan Technology Co., Ltd. (Shanghai, China). GO was purchased from Jining Leader Nano Technology Co., Ltd. (Jining, China). Tetraethyl orthosilicate (TEOS, 98%) was purchased from Mreda Technology Co., Ltd. (Beijing, China). Formic acid (HPLC grade), methanol (HPLC grade), ethanol (HPLC grade), acetone (HPLC grade), acetonitrile (HPLC grade), acetic acid (HPLC grade), humic acid (HA, 90%), glucose (98%), fructose (98%), and sucrose (98%) were purchased from Kemiou Chemical Reagent Co., Ltd. (Tianjin, China). Ultrapure water (prepared by Purelab Classic Elga, High Wycombe, UK) was used in all experiments.

The characterization of the composite material’s morphology was performed using scanning electron microscopy (SEM, QUANTA 430, FEI) and transmission electron microscopy (TEM, TECNAI G2 20, FEI), both instruments located in Hillsboro, OR, USA. Fourier transform infrared (FTIR) spectroscopy (NICOLET 5700, Thermo Electron, Waltham, MA, USA) was employed for surface functional group identification, using the KBr pellet method at a resolution of 4 cm^−1^ to ensure optimal balance between resolution and signal-to-noise ratio. X-ray diffraction (XRD) analysis was conducted with a D8 Advance XRD analyzer (Bruker, Germany), using Cu Kα radiation at 40 kV and 40 mA to determine the crystal structure. Furthermore, the surface attributes, including porosity and specific surface area, were quantified via the Brunauer–Emmett–Teller (BET) technique, utilizing a Quantachrome Autosorb-iQ system (Boynton Beach, FL, USA), to evaluate the adsorbent’s efficacy. Thermogravimetric analysis (TGA) of oven-dried powder samples was performed on a thermogravimetric analyzer (Q500, TA, New Castle, DE, USA). The magnetic behavior of the sample was characterized at ambient temperature utilizing a magnetic property measurement system (MPMS SQUID XL, Quantum Design, San Diego, CA, USA) to acquire the hysteresis curve. A Shimadzu LC-10AT HPLC system with an RF-20A fluorescence detector (FLD, Kyoto, Japan) was used for analysis.

### 3.2. Synthesis of MNCGC

#### 3.2.1. Synthesis of Silica-Coated Fe_3_O_4_

Silica-coated Fe_3_O_4_ nanoparticles were synthesized using a combination of alkaline coprecipitation and the sol-gel technique. Initially, ferric chloride hexahydrate (FeCl_3_·6H_2_O) and ferrous sulfate heptahydrate (FeSO_4_·7H_2_O) were solubilized in deionized water, maintaining a 2:1 molar ratio, followed by continuous stirring in a nitrogen environment. Ammonia water was slowly added until the pH reached approximately 10. Then, the solution was heated at 80 °C for 30 min. The Fe_3_O_4_ nanoparticles were separated using a magnet and subjected to repeated washing with both water and ethanol. Subsequently, they were dried under vacuum conditions at 50 °C for a duration of 12 h. Following this drying process, 0.5 g of the Fe_3_O_4_ nanoparticles were dispersed into a solution comprising 40 mL of water and 160 mL of ethanol, preparing them for the next stage of synthesis. The mixture was then continuously stirred and sonicated for 30 min to allow uniform dispersion. A total of 0.5 mL of TEOS and 4 mL of NH_3_·H_2_O were slowly added dropwise to the solution. After stirring for 6 h at 30 °C, the solid phase and liquid phase were separated with a magnet. Finally, the obtained solid was washed three times with absolute ethanol and then vacuum-dried at 50 °C for 12 h.

#### 3.2.2. Synthesis of MNCGC

An amount of 1 g of CS was dissolved in 120 mL of acetic acid solution (2%, *v*/*v*), and the mixture was sonicated for 5 min. Then, 0.5 g Fe_3_O_4_@SiO_2_ was added to the completely dissolved CS solution, and the mixture was vigorously stirred at 30 °C for 2 h. Next, 2 g GO, 5 mL glutaraldehyde (50%), and 5 mL ammonia water (25%) were added, followed by stirring at 80 °C for 2 h to complete the crosslinking and amidation reactions. Finally, the solid was collected with a magnet, washed with water and ethanol several times, and vacuum-dried at 50 °C for 12 h. A total of 0.5 g of the above-described dried product and 5 g of β-CD were added to 120 mL of water. The mixture was sonicated with vigorous stirring for 10 min and then stirred at 60 °C for 5 h. The solid was separated, collected by a magnet, washed several times with water, and vacuum-dried at 50 °C for 12 h to obtain the final product, MNCGC.

### 3.3. MSPE Procedure

**Figure 15:**
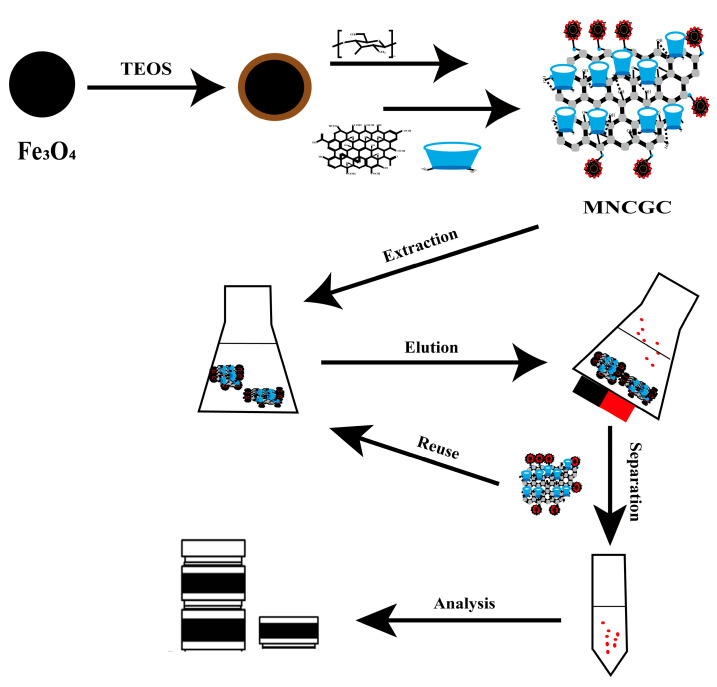
Synthesis route of MNCGC and a typical MSPE process.

The MSPE process is shown in Figure 15. A certain amount of MNCGC was added to 30 mL of mixed solution containing 10 μg/L BP pollutants and uniformly dispersed. Then, the mixture was placed in a gas bath thermostatic shaker, and shaking extraction was performed at 200 rpm and 30 °C for 60 min. After the extraction was complete, the solid phase and liquid phase were separated by a magnet, the supernatant was discarded, and 5 mL of eluent was added and dispersed evenly. The mixture was then placed in a gas bath thermostatic shaker, and shaking elution was performed at 200 rpm and 30 °C for 60 min. After elution, MNCGC was separated by a magnet, and the supernatant was slowly dried with nitrogen at room temperature. The residue was dissolved in methanol to a volume of 500 μL. The sample was vortexed for 30 s, filtered through a 0.22-μm organic phase filter, and finally measured with HPLC–FLD.

### 3.4. HPLC Analysis

BPA, BPB, BPF, and BPAF were separated and analyzed by a Shimadzu LC-10AT HPLC system with an Agilent C18 column (250 × 4.6 mm, 5 μm) and a Shimadzu RF-20A fluorescence detector. Isocratic elution was performed using water and methanol (30:70, *v*/*v*) with a flow rate of 0.8 mL/min. The excitation and emission wavelengths were set to 227 nm and 313 nm, respectively, and the column temperature was controlled at 30 °C. The injection volume was 20 μL.

## 4. Conclusions

In this study, a novel magnetic adsorbent (MNCGC) was developed via a multistep preparation method using various functional monomers (CS, GO, and β-CD). Fe_3_O_4_ was used as the magnetic core, and SiO_2_ was coated on Fe_3_O_4_ to enhance the stability and dispersion of Fe_3_O_4_. CS was then introduced onto the surface of silica-coated Fe_3_O_4_ by crosslinking. The abundant amino groups on CS underwent amidation reactions with the carboxyl groups on the GO surface to achieve grafting. Then, β-CD was connected through hydrogen bonds, hydrophobic bonds, and van der Waals forces to form the final product, MNCGC. MNCGC was characterized by SEM, TEM, EDS, FTIR, XRD, BET, TGA, and VSM. The results showed that MNCGC was successfully prepared with a favorable morphology and crystal structure. The specific surface area was 91.83 m^2^/g, the pore size was 12.08 nm, and the magnetization was 16.2 emu/g. Separation of MNCGC was achieved by applying an external magnetic field. Next, MNCGC was used as an adsorbent in MSPE, which was coupled with HPLC–FLD analysis. An analytical method that can simultaneously determine the four target BP pollutants in environmental water and food samples was established. The extraction and elution conditions were optimized; interference tests were performed using HA, glucose, fructose, and sucrose as interference factors; and the established method was methodologically evaluated. The results showed that the interference factors had basically no impact. This method featured a favorable linearity range, low LOD (0.01–0.02 μg/L), low LOQ (0.03–0.05 μg/L), and good recycling performance. Finally, seven environmental and food samples were analyzed under both blank and spiked conditions, and the results were satisfactory. Therefore, this study introduces MNCGC, a novel material, and demonstrates its application through a new analytical method for the efficient detection of trace bisphenol pollutants in environmental and food samples.

## Figures and Tables

**Table 1:** Methodological evaluation of the MSPE–HPLC–FLD method for the determination of four bisphenol (BP) pollutants.

Compound	Linearity Range (μg/L)	*R*	LOQ (μg/L)	LOD (μg/L)	RSD (*n* = 6)	RSD (*n* = 3)
BPA	0.03–100	0.9999	0.03	0.01	2.13	4.34
BPB	0.05–100	0.9995	0.05	0.01	2.57	6.81
BPF	0.05–100	0.9998	0.05	0.02	3.41	5.43
BPAF	0.04–100	0.9989	0.04	0.01	2.79	7.17

**Table 2:** Sampling coordinates of environmental water samples.

	Sampling Points	Coordinate
Fuhe River	S1	115°30′59″ E, 38°52′10″ N
	S2	115°34′6″ E, 38°52′1″ N
Baiyang Lake	S3	115°96′457″ E, 38°84′125″ N
	S4	115°95′141″ E, 38°84′88″ N

**Table 3:** Environmental and food sample analysis results.

Samples and Sampling Points	BPA (μg/L)	Recovery (%)	BPB (μg/L)	Recovery (%)	BPF (μg/L)	Recovery (%)	BPAF (μg/L)	Recovery (%)
Fuhe River								
S1	ND ^a^		0.16		0.11		ND	
5 (μg/L)		93.4 ^b^ ± 1.5 ^c^		95.1 ± 1.7		89.3 ± 2.3		90.2 ± 2.1
10 (μg/L)		94.1 ± 1.4		96.3 ± 2.1		89.7 ± 1.8		89.7 ± 1.8
S2	0.09		0.17		ND		0.17	
5 (μg/L)		92.7 ± 2.1		96.2 ± 1.4		89.9 ± 1.5		92.5 ± 1.2
10 (μg/L)		93.3 ± 1.8		96.6 ± 1.6		90.3 ± 1.7		93.1 ± 1.9
Baiyang Lake								
S3	0.05		0.15		ND		ND	
5 (μg/L)		93.1 ± 2.3		95.2 ± 1.3		90.5 ± 1.4		91.8 ± 1.4
10 (μg/L)		92.9 ± 1.2		96.0 ± 1.8		91.8 ± 2.1		92.4 ± 2.2
S4	0.06		0.14		0.07		ND	
5 (μg/L)		93.2 ± 1.1		95.9 ± 1.1		91.3 ± 2.0		90.4 ± 1.7
10 (μg/L)		93.4 ± 1.6		94.2 ± 1.4		92.1 ± 3.2		93.1 ± 2.0
Food								
Walnut milk	0.18		ND		4.92		3.53	
5 (μg/L)		95.1 ± 2.7		95.1 ± 1.6		99.7 ± 2.7		100.8 ± 1.6
10 (μg/L)		96.3 ± 1.9		93.9 ± 2.8		102.3 ± 1.9		103.0 ± 2.4
Peach juice	0.19		0.22		ND		ND	
5 (μg/L)		94.1 ± 1.6		96.3 ± 2.0		91.4 ± 1.6		92.4 ± 2.2
10 (μg/L)		94.6 ± 2.3		96.7 ± 1.5		89.1 ± 2.7		93.1 ± 1.9
Orange juice	ND		0.96		0.22		ND	
5 (μg/L)		92.4 ± 3.1		97.2 ± 1.3		93.8 ± 1.3		89.9 ± 1.4
10 (μg/L)		93.3 ± 2.2		98.0 ± 2.2		95.6 ± 1.8		91.1 ± 1.7

^a^ ND: Not Detected. ^b^ Mean of three determinations. ^c^ Mean of three determinations.

**Table 4:** Comparison of the proposed method with other reported methods.

Method	Adsorbent	Analytes	LODs (μg/L)	Recovery (%)	Reference
MSPE–HPLC–MS ^a^	Fe_3_O_4_@COF	Bisphenols	0.001–0.078	64.8–92.8	[25]
LPMD ^b^–HPLC–UV ^c^	Ferrofluids	Bisphenols	0.09–0.17	94.5–102.1	[34]
SPE ^d^–HPLC–DAD ^e^	HPCSs	Bisphenols	0.05–0.53	89.6–111.5	[35]
MSPE–HPLC–UV	Gn-MNPs	Bisphenols	0.011–0.017	93.8–101.1	[26]
MSPE–HPLC–FLD	MNCGC	Bisphenols	0.01–0.02	90.9–94.0	This study

^a^ Mass spectrometry. ^b^ Dispersive solid-phase extraction. ^c^ Ultraviolet detection. ^d^ Solid-phase extraction. ^e^ Diode array detection.

## Data Availability

Data will be made available on request.

## References

[1] Colborn T., vom Saal F.S., Soto A.M. (1993). Developmental effects of endocrine-disrupting chemicals in wildlife and humans. Environ. Health Perspect..

[2] Sofen L.E., Furst A.L. (2019). Perspective—Electrochemical sensors to monitor endocrine disrupting pollutants. J. Electrochem. Soc..

[3] Dhanjai, Sinha A., Wu L., Lu X., Chen J., Jain R. (2018). Advances in sensing and biosensing of bisphenols: A review. Anal. Chim. Acta.

[4] Yin H., Zhou Y., Cui L., Liu X., Ai S., Zhu L. (2011). Electrochemical oxidation behavior of bisphenol A at surfactant/layered double hydroxide modified glassy carbon electrode and its determination. J. Solid State Electrochem..

[5] Michałowicz J. (2014). Bisphenol A—Sources, toxicity and biotransformation. Environ. Toxicol. Pharmacol..

[6] Liang X., Yin N., Liang S., Yang R., Liu S., Lu Y., Jiang L., Zhou Q., Jiang G., Faiola F. (2020). Bisphenol A and several derivatives exert neural toxicity in human neuron-like cells by decreasing neurite length. Food Chem. Toxicol..

[7] Kim J.J., Kumar S., Kumar V., Lee Y.M., Kim Y.S., Kumar V. (2019). Bisphenols as a legacy pollutant, and their effects on organ vulnerability. Int. J. Environ. Res. Public Health.

[8] Santoro A., Chianese R., Troisi J., Richards S., Nori S.L., Fasano S., Guida M., Plunk E., Viggiano A., Pierantoni R. (2019). Neuro-toxic and reproductive effects of BPA. Curr. Neuropharmacol..

[9] Liu B., Lehmler H.J., Sun Y., Xu G., Liu Y., Zong G., Sun Q., Hu F.B., Wallace R.B., Bao W. (2017). Bisphenol A substitutes and obesity in US adults: Analysis of a population-based, cross-sectional study. Lancet Planet. Health.

[10] Chin K.Y., Pang K.L., Mark-Lee W.F. (2018). A review on the effects of bisphenol A and its derivatives on skeletal health. Int. J. Med. Sci..

[11] Catenza C.J., Farooq A., Shubear N.S., Donkor K.K. (2020). A targeted review on fate, occurrence, risk and health implications of bisphenol analogues. Chemosphere.

[12] Liu D., Liu J., Guo M., Xu H., Zhang S., Shi L., Yao C. (2016). Occurrence, distribution, and risk assessment of alkylphenols, bisphenol A, and tetrabromobisphenol A in surface water, suspended particulate matter, and sediment in Taihu Lake and its tributaries. Mar. Pollut. Bull..

[13] Liu A.F., Qu G.B., Yu M., Liu Y.W., Shi J.B., Jiang G.B. (2016). Tetrabromobisphenol-A/S and nine novel analogs in biological samples from the Chinese Bohai Sea: Implications for trophic transfer. Environ. Sci. Technol..

[14] Song S., Ruan T., Wang T., Liu R., Jiang G. (2012). Distribution and preliminary exposure assessment of bisphenol AF (BPAF) in various environmental matrices around a manufacturing plant in China. Environ. Sci. Technol..

[15] Liu J., Zhang L., Lu G., Jiang R., Yan Z., Li Y. (2021). Occurrence, toxicity and ecological risk of Bisphenol A analogues in aquatic environment—A review. Ecotoxicol. Environ. Saf..

[16] Lee S., Liao C., Song G.J., Ra K., Kannan K., Moon H.B. (2015). Emission of bisphenol analogues including bisphenol A and bisphenol F from wastewater treatment plants in Korea. Chemosphere.

[17] Zhang H., Zhang Y., Li J., Yang M. (2019). Occurrence and exposure assessment of bisphenol analogues in source water and drinking water in China. Sci. Total Environ..

[18] Wang H., Liu Z.H., Tang Z., Zhang J., Yin H., Dang Z., Wu P.X., Liu Y. (2020). Bisphenol analogues in Chinese bottled water: Quantification and potential risk analysis. Sci. Total Environ..

[19] Huang C., Wu L.H., Liu G.Q., Shi L., Guo Y. (2018). Occurrence and ecological risk assessment of eight endocrine-disrupting chemicals in urban river water and sediments of South China. Arch. Environ. Contam. Toxicol..

[20] Chen X.W., Zhao J.L., Liu Y.S., Jiang Y.X., Yang Y.Y. (2016). Occurrence and ecological risks of hormonal activities in the middle and lower reaches of Yangtze River. Asian J. Ecotoxicol..

[21] Chen D., Kannan K., Tan H., Zheng Z., Feng Y.L., Wu Y., Widelka M. (2016). Bisphenol analogues other than BPA: Environmental occurrence, human exposure, and toxicity—A review. Environ. Sci. Technol..

[22] Cao P., Zhong H.N., Qiu K., Li D., Wu G., Sui H.X., Song Y. (2021). Exposure to bisphenol A and its substitutes, bisphenol F and bisphenol S from canned foods and beverages on Chinese market. Food Control.

[23] Lian L., Lv J., Wang X., Lou D. (2018). Magnetic solid–phase extraction of tetracyclines using ferrous oxide coated magnetic silica microspheres from water samples. J. Chromatogr. A.

[24] De Souza K.C., Andrade G.F., Vasconcelos I., de Oliveira Viana I.M., Fernandes C., de Sousa E.M.B. (2014). Magnetic solid-phase extraction based on mesoporous silica-coated magnetic nanoparticles for analysis of oral antidiabetic drugs in human plasma. Mater. Sci. Eng. C.

[25] Chen L., He Y., Lei Z., Gao C., Xie Q., Tong P., Lin Z. (2018). Preparation of core-shell structured magnetic covalent organic framework nanocomposites for magnetic solid-phase extraction of bisphenols from human serum sample. Talanta.

[26] Wu Y., Chen C., Zhou Q., Li Q.X., Yuan Y., Tong Y., Wang H., Zhou X., Sun Y., Sheng X. (2019). Polyamidoamine dendrimer decorated nanoparticles as an adsorbent for magnetic solid-phase extraction of tetrabromobisphenol A and 4-nonylphenol from environmental water samples. J. Colloid Interface Sci..

[27] Giannakas A., Patsaoura A., Barkoula N.M., Ladavos A. (2017). A novel solution blending method for using olive oil and corn oil as plasticizers in chitosan based organoclay nanocomposites. Carbohydr. Polym..

[28] Wang J., Zhang J., Han L., Wang J., Zhu L., Zeng H. (2021). Graphene-based materials for adsorptive removal of pollutants from water and underlying interaction mechanism. Adv. Colloid Interface Sci..

[29] Wang Z., Zhang P., Hu F., Zhao Y., Zhu L. (2017). A crosslinked β-cyclodextrin polymer used for rapid removal of a broad-spectrum of organic micropollutants from water. Carbohydr. Polym..

[30] Araj S.K., Szeleszczuk Ł. (2023). A Review on Cyclodextrins/Estrogens Inclusion Complexes. Int. J. Mol. Sci..

[31] Yamaura M., Camilo R.L., Sampaio L.C., Macêdo M.A., Nakamura M., Toma H.E. (2004). Preparation and characterization of (3-aminopropyl)triethoxysilane-coated magnetite nanoparticles. J. Magn. Magn. Mater..

[32] Dong A., Lan S., Huang J., Wang T., Zhao T., Xiao L., Wang W., Zheng X., Liu F., Gao G. (2011). Modifying Fe_3_O_4_-functionalized nanoparticles with N-halamine and their magnetic/antibacterial properties. ACS Appl. Mater. Interfaces.

[33] Yang K., Xing B. (2010). Adsorption of organic compounds by carbon nanomaterials in aqueous phase: Polanyi theory and its application. Chem. Rev..

[34] Yang D., Li X., Meng D., Yang Y. (2018). Carbon quantum dots-modified ferrofluid for dispersive solid-phase extraction of phenolic compounds in water and milk samples. J. Mol. Liq..

[35] Zhang Z., Zhang J., Wang Y., Tong Y., Zhang L. (2017). Controlled synthesis of hollow porous carbon spheres for enrichment and simultaneous determination of nine bisphenols from real samples. Talanta.

